# PM_10_ and gaseous pollutants trends from air quality monitoring networks in Bari province: principal component analysis and absolute principal component scores on a two years and half data set

**DOI:** 10.1186/1752-153X-8-14

**Published:** 2014-02-21

**Authors:** Pierina Ielpo, Vincenzo Paolillo, Gianluigi de Gennaro, Paolo Rosario Dambruoso

**Affiliations:** 1 Water Research Institute - National Research Council, Bari division, viale de Blasio, 5 70132 Bari, Italy; 2 Chemistry Department, Bari University, via Orabona, 4 70126 Bari, Italy; 3 Institute of Atmospheric Sciences and Climate, Lecce division, str Lecce-Monteroni Km 1.2, 73100 Lecce, Italy

**Keywords:** PM_10_, Gaseous pollutants, Air monitoring stations, PCA, APCS, Source apportionment

## Abstract

**Background:**

The chemical composition of aerosols and particle size distributions are the most significant factors affecting air quality. In particular, the exposure to finer particles can cause short and long-term effects on human health. In the present paper PM_10_ (particulate matter with aerodynamic diameter lower than 10 μm), CO, NO_x_ (NO and NO_2_), Benzene and Toluene trends monitored in six monitoring stations of Bari province are shown. The data set used was composed by bi-hourly means for all parameters (12 bi-hourly means per day for each parameter) and it’s referred to the period of time from January 2005 and May 2007. The main aim of the paper is to provide a clear illustration of how large data sets from monitoring stations can give information about the number and nature of the pollutant sources, and mainly to assess the contribution of the traffic source to PM_10_ concentration level by using multivariate statistical techniques such as Principal Component Analysis (PCA) and Absolute Principal Component Scores (APCS).

**Results:**

Comparing the night and day mean concentrations (per day) for each parameter it has been pointed out that there is a different night and day behavior for some parameters such as CO, Benzene and Toluene than PM_10_. This suggests that CO, Benzene and Toluene concentrations are mainly connected with transport systems, whereas PM_10_ is mostly influenced by different factors.

The statistical techniques identified three recurrent sources, associated with vehicular traffic and particulate transport, covering over 90% of variance. The contemporaneous analysis of gas and PM_10_ has allowed underlining the differences between the sources of these pollutants.

**Conclusions:**

The analysis of the pollutant trends from large data set and the application of multivariate statistical techniques such as PCA and APCS can give useful information about air quality and pollutant’s sources. These knowledge can provide useful advices to environmental policies in order to reach the WHO recommended levels.

## Background

The knowledge of chemical composition and sources of air polluted is demanded in any program aimed at controlling the levels of pollutants in order to evaluate and reduce their impact on human health.

The inhalation of air polluted, in fact, with particulate matter (PM_10_) and or irritant gases such as NO_2_ and SO_2_ is associated with both short-term and long term health effects, most of which impact on respiratory and cardiovascular system [1]. For example the atmospheric concentrations of NO_2_ have been linked to the deaths of severely asthmatic patients in Barcelona [2], child asthma cases in Toronto and Southern California [3,4], heart rate dysfunction in Taiwan and Switzerland [5,6], and ischemic heart disease in elderly residents of French cities [7]. Similar examples can be chosen to illustrate the damaging effects of PM_10_ inhalation, whether it be asthma in Madrid or Sydney [8,9] or all-cause mortality (especially stroke) in Boston [10].

The federal Clean Air Act Amendments of 1990 mandate that the U.S. EPA determine a set of urban hazardous air pollutants (PAHs, or ‘air toxics’) that potentially pose the greatest risks in urban areas, in terms of contribution to population health risk. The current set of 188 PAHs includes toxic metals and volatile organic compounds (VOCs). The U.S. EPA identified 33 urban PAHs based on emissions and toxicities in a 1995 ranking analysis [11] and developed concurrent monitoring and modelling programs to evaluate potential exposures and risks to these top-ranked 33 PAHs. Developing effective control strategies to reduce population exposure to certain PAHs requires identifying sources and quantifying their contributions to the mixture of PAHs and the associated health risks. One approach is to use receptor-based source apportionment models to distinguish sources. Most source apportionment studies focus on analysing either VOCs [12,13] or fine particle (PM_2.5_) mass [14-16]. Only few studies used source apportionment modelling to identify common sources of both VOCs and PM_2.5_. In other source apportionment studies that included both non-organic trace elements on PM and gaseous pollutants [17-20], the gaseous species usually were non-VOCs (such as CO, SO_2_, and NO).

In recent years, there has been an increased interest in the application of chemometrics [21] to different environmental research fields, ranging from water to air pollution and cultural heritage [22-25]. One aspect of the application of chemometrics to environmental pollution research is often referred to as source apportionment, receptor modelling and/or mixture analysis discipline. Recent examples of such work can be found in Europe [26,27], the US [28,29] and Asia [30,31]. In the fields of pollution sciences (air or water), source apportionment models aim to re-construct the emissions from different sources of pollutants based on ambient data registered at monitoring sites [32].

In the present paper a bihourly data set of PM_10_, CO, NO_x_, Benzene and Toluene collected in six air quality monitoring stations of Bari territory from January 2005 to May 2007 is used. The main aim of this paper is to provide a clear illustration of how large data sets from monitoring stations can give information about the number and nature of the pollutant sources, and mainly to assess the contribution of the traffic source to PM_10_ concentration level by using multivariate statistical techniques.

These knowledge could provide useful advices to environmental policies in order to reach the WHO recommended levels. In fact legislative efforts to reduce the health effects of air pollutants are currently being applied throughout the developed world, with the imposition of averaged limit values which vary for different pollutants. In the case of PM_10_, the World Health Organization has recommended progressive achievement of four pollution thresholds which cascade down through three Interim Targets (IT1 ¼ 70 μg/m^3^; IT2 ¼ 50 μg/m^3^; IT3 ¼ 30 μg/m^3^) to reach the ultimate objective: an Air Quality Guideline (AQG) annual mean of just 20 μg m^3^ PM_10_[33,34]. Moreover considering the latest Italian law [35,36] for PM_10_ the annual limit value is 40 μg/m^3^, while the daily limit value is 50 μg/m^3^; for NO_x_ the annual limit value is 40 μg/m^3^, while hourly limit value is 200 μg/m^3^; for Benzene the annual limit value is 5 μg/m^3^ and for CO the 8 hour mean limit value is 10 mg/m^3^.

## Results and discussion

In the Table 1 the basic statistics for each site have been summarized. Among all the available sampling sites, only those with the number of data not less than 5000 were used, considering only days with complete data (12 daily data). High variability is explained by the long range of the period (2.5 years). Pollutants concentrations were reported as μg/m^3^, except for CO which is expressed as mg/m^3^.

**Table 1:** Descriptive statistics for each parameter in each site

	**Viale Archimede, Bari (urban site)**			**San Nicola sport stadium, Bari (suburban site)**			**Viale King, Bari (urban site)**		
	**Data**	**Mean**	**STD. DEV.**	**Data**	**Mean**	**STD. DEV.**	**Data**	**Mean**	**STD. DEV.**
***NOx***	7456	42.15	41.69	6648	24.65	32.82	7008	61.64	57.88
***Benzene***	7456	1.49	1.38	6648	1.08	1.48	7008	1.56	1.62
***Toluene***	7456	4.18	4.07	6648	2.40	4.02	7008	4.97	7.46
***CO***	7456	0.45	0.35	6648	0.44	0.18	7008	0.58	0.41
***PM10***	7456	29.71	14.36	6648	31.59	20.26	7008	25.66	16.08
	**Altamura, prov. of Bari (urban site)**			**Andria, prov. of Bari (urban site)**			**Monopoli, prov. of Bari (urban site)**		
	**Data**	**Mean**	**STD. DEV.**	**Data**	**Mean**	**STD. DEV.**	**Data**	**Mean**	**STD. DEV.**
***NOx***	5136	46.12	40.08	5568	43.89	42.79	5244	50.65	47.60
***Benzene***	5136	1.83	1.62	5568	1.66	0.95	5244	1.98	1.06
***Toluene***	5136	6.74	9.27	5568	4.72	4.47	5244	4.98	4.18
***CO***	5136	0.43	0.32	5568	0.77	0.73	5244	0.46	0.34
***PM10***	5136	27.83	20.83	5568	22.00	14.63	5244	36.11	21.91

**Figure 1:**
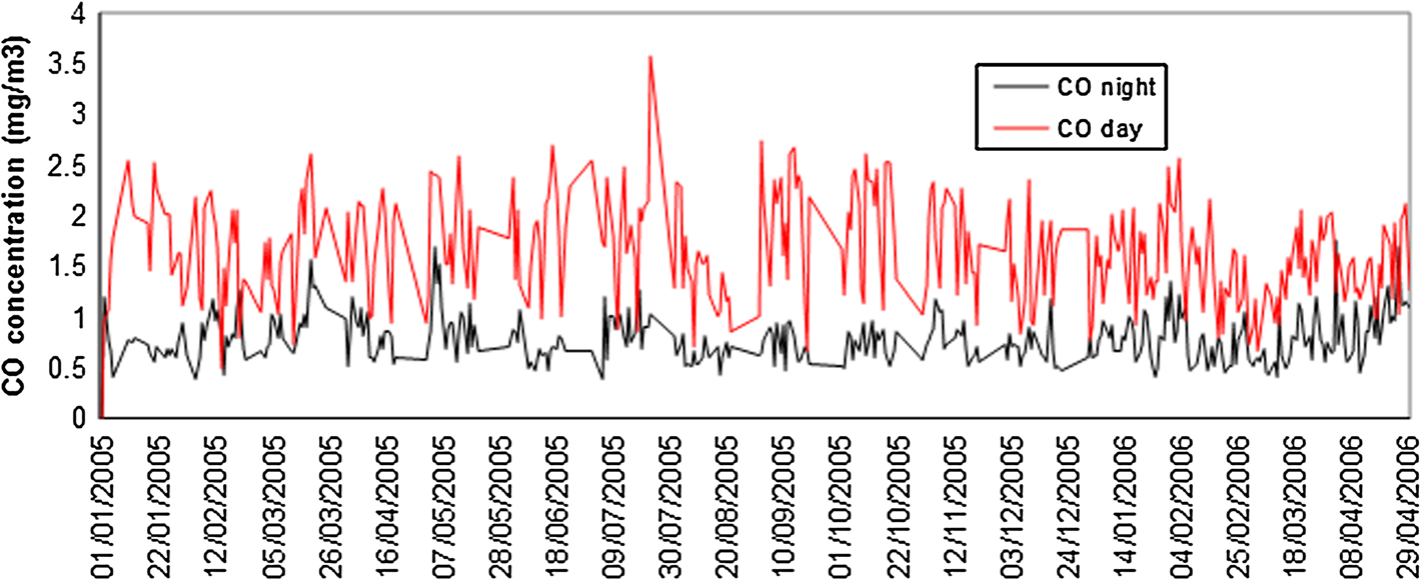
CO concentrations daily and night trend for all the data collected in Viale Archimede.

**Figure 2:**
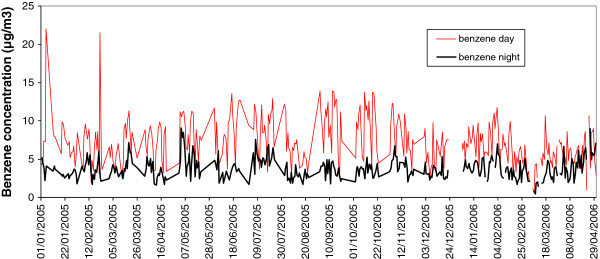
Benzene concentrations daily and night trend for all the data collected in Viale Archimede.

**Figure 3:**
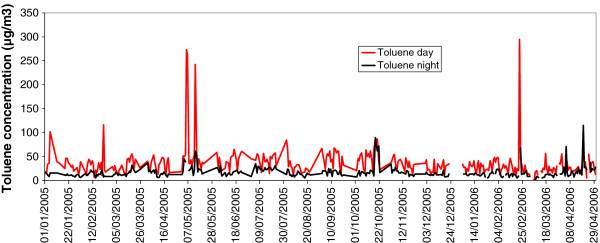
Toluene concentrations daily and night trend for all the data collected in Viale Archimede.

**Figure 4:**
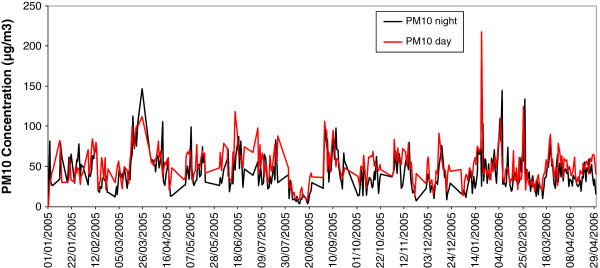
PM10 concentrations daily and night trend for all the data collected in Viale Archimede.

From data collected, night and daily mean concentrations (per day) for each parameter have been obtained. Night and daily mean values have been plotted for each parameter and graphics, as Figures 1, 2, 3 and 4 shown, have been obtained for each sampling site.

Observing the Figures 1, 2, 3 and 4 shown as example, parameters such as CO, Benzene and Toluene show different trend between night and daily values, whit daily mean values bigger than night ones. In particular for the data shown in Figures 1, 2 and 3 the percentage ratio between (daily mean - night mean) and daily mean for CO, Benzene and Toluene is 53%, 49%, 54% respectively. Considering Toluene trend shown in Figure 3 it is possible to note for some days, e. g. 05/05/2005 or 22/02/2006, very high daily mean values on the contrary of Benzene ones shown in Figure 2. The reason is due to the presence of another pollution source affecting the monitoring site, probably identifiable in the painting of pedestrian crossing and road stripes.

Considering the PM_10_ night and dilay mean concentrations (Figure 4) it’s possible to note that they don’t show a clear difference between day and night: in fact the ratio for PM_10_ is 16%. Moreover for some days, e. g. 25/03/2005 and 06/02/2006, the thermodynamic conditions in the planetary boundary layer (PBL) adversely affect pollutants dispersion leading to PM_10_ night values bigger than daily ones, in spite of emission sources reduction during the night.

The different night and daily behavior suggests that parameters such as CO, Benzene and Toluene are mainly connected with transport systems, whereas PM_10_ is mostly influenced by different factors.

The parameters trends shown in Figures 1, 2, 3 and 4, related to Viale Archimede data, are similar to ones of the other sites. So the different behaviour between PM_10_ and the other parameters (CO, Benzene, Toluene) can be considered common to the whole area investigated: Bari and Bari province.

Moreover, as we have shown in a previous papers [37], the results obtained both by automatic monitoring stations and sampling campaigns in several sites of Apulia region, suggest that the PM_10_ amount monitored in this area presents a common contribution also among monitoring stations located at 70 km far each other: the common contribution apparently does not depend from local sources. Moreover in the reference 37 we pointed out that PM_10_ concentrations do not show a seasonal trend, contrary to the PM_10_ trend shown in the towns of North Italy [38,39].

In order to identify the pollutant sources that contribute to PM_10_ concentrations and try to distinguish the contribution of local sources, such as vehicular traffic, as respect to “a common regional source” (that is re-suspended matter, dust intrusions, calcium carbonate source), APCS model has been applied to the data collected.

According to the criteria described in the methods section we have chosen the ODV90 one, revealing that three components are necessary and sufficient to run properly the model.

In Table 2 the loading’s values for the PC analysis applied to the data collected in all the sites during January 2007 are shows as example. Three factors explain almost the 92% of the total variance of data for all the sites. Factor loadings are used to obtain information about source’s profiles. The first factor (or first principal component, PC1) accounting for a percentage of the total variance ranging between 40% and 51% was dominated by high loading values of Benzene, Toluene and CO, or by NO_x_ and CO depending on the sites; the second factor (or second principal component, PC2), accounting for a percentage ranging between 24% and 31% of the total variance, is dominated by PM_10_ or by Benzene and Toluene, while the third factor explaining a percentage ranging between 21% and 25% of the total variance had high loadings values for Benzene and Toluene or PM_10_.

**Table 2:** Principal Component Analysis loadings for all the sites investigated during January 2007

	**Viale Archimede, Bari (urban site)**			**San Nicola sport stadium, Bari (suburban site)**			**Viale King, Bari (urban site)**		
	**PC1**	**PC2**	**PC3**	**PC1**	**PC2**	**PC3**	**PC1**	**PC2**	**PC3**
***NOx***	0.14	0.84	0.01	0.90	0.02	0.02	0.09	0.84	0.04
***Benzene***	0.75	0.06	0.13	0.07	0.77	0.13	0.70	0.14	0.14
***Toluene***	0.75	0.09	0.09	0.53	0.18	0.12	0.84	0.09	0.04
***CO***	0.67	0.23	0.00	0.48	0.29	0.10	0.30	0.43	0.15
***PM10***	0.06	0.01	0.93	0.06	0.12	0.82	0.08	0.05	0.86
***Eigenvalues***	2.37	1.22	1.17	2.04	1.37	1.19	2.02	1.55	1.23
**% *variance explained***	47.3	24.5	23.4	40.9	27.5	23.9	40.5	31.1	24.7
	**Altamura, prov. of Bari (urban site)**			**Andria, prov. of Bari (urban site)**			**Monopoli, prov. of Bari (urban site)**		
	**PC1**	**PC2**	**PC3**	**PC1**	**PC2**	**PC3**	**PC1**	**PC2**	**PC3**
***NOx***	0.84	0.03	0.11	0.87	0.11	0.01	0.91	0.00	0.03
***Benzene***	0.22	0.10	0.66	0.33	0.47	0.17	0.68	0.19	0.05
***Toluene***	0.34	0.27	0.31	0.24	0.61	0.13	0.05	0.01	0.95
***CO***	0.89	0.00	0.09	0.86	0.12	0.01	0.90	0.03	0.02
***PM10***	0.01	0.94	0.05	0.01	0.06	0.93	0.02	0.96	0.01
***Eigenvalues***	2.30	1.34	1.22	2.31	1.38	1.24	2.55	1.19	1.05
**% *variance explained***	46.0	26.8	24.5	46.1	27.6	24.8	51.1	23.9	21.0

Applying PCA on all data set generally we found that for each sampling site one of the three factors is characterized by high loading values of PM_10_, the other two factors are characterized by high loading values of NO_x_ , CO, Benzene and Toluene.

**Figure 5:**
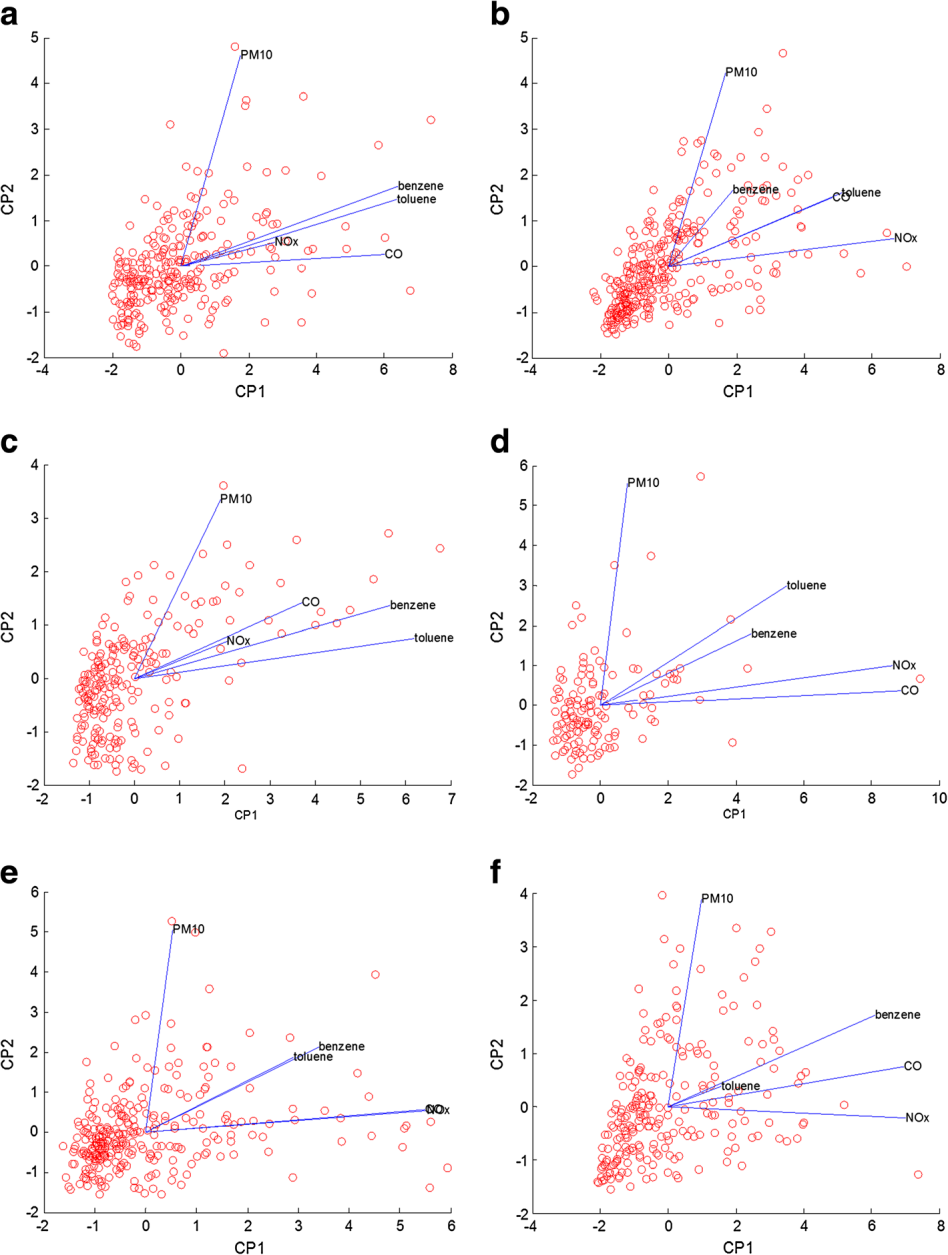
**Scores and Loading plots in the plane of first and second Principal Component (PC1 and PC2) obtained for the data set of parameters collected during January 2007 in viale Archimede (a)**, **S. Nicola sport stadium (b)**, **viale King (c)**, **Altamura (d)**, **Andria (e) and Monopoli (f) sites.**

Observing Figure 5 it’s possible to note that PM_10_ is the dominant parameter on the second component with high loading values.

In order to identify the three sources the Absolute Principal Component Scores model has been applied to data sets. In the Tables 3 and 4 the source’s profiles for each monitoring station are shown as example. The source’s profiles shown are the average, obtained during the Summer 2006 and Winter 2006, of the monthly profiles.

**Table 3:** **APCS source**’**s profiles for the data collected during the Summer season 2006 in all the monitoring stations investigated**

	**Viale Archimede, Bari (urban site)**			**San Nicola sport stadium, Bari (suburban site)**			**Viale King**, **Bari (urban site)**		
	**I Source**	**II Source**	**III Source**	**I Source**	**II Source**	**III Source**	**I Source**	**II Source**	**III Source**
***NOx***	29.25	6.56	21.07	13.15	3.80	6.89	20.38	3.74	23.74
***Benzene***	0.468	0.239	0.515	0.066	0.264	0.353	0.521	0.139	0.450
***Toluene***	1.688	0.952	1.685	0.794	0.409	0.402	1.223	0.943	1.570
***CO***	0.121	0.045	0.110	0.054	0.066	0.044	0.086	0.049	0.726
***PM10***	3.91	23.06	4.96	3.35	25.45	2.57	3.29	24.46	1.77
	**Altamura, prov. of Bari (urban site)**			**Andria, prov. of Bari (urban site)**			**Monopoli, prov. of Bari (urban site)**		
	**I Source**	**II Source**	**III Source**	**I Source**	**II Source**	**III Source**	**I Source**	**II Source**	**III Source**
***NOx***	12.40	2.28	8.35	25.87	1.26	13.56	18.02	2.71	18.05
***Benzene***	0.100	0.056	0.368	0.691	0.122	0.246	0.848	0.278	0.276
***Toluene***	0.278	0.358	1.466	1.501	0.720	1.277	1.649	0.525	1.392
***CO***	0.116	0.039	0.081	0.340	0.014	0.133	0.187	0.033	0.060
***PM10***	1.19	40.45	6.92	1.74	17.69	1.07	3.21	38.12	1.29

Parameter’s unit is ug/m^3^.

**Table 4:** **APCS Source**’**s profiles for the data collected during the Winter season 2006 in all the monitoring stations investigated**

	**Viale Archimede, Bari (urban site)**			**San Nicola sport stadium, Bari (suburban site)**			**Viale King, Bari (urban site)**		
	**I Source**	**II Source**	**III Source**	**I Source**	**II Source**	**III Source**	**I Source**	**II Source**	**III Source**
***NOx***	35.52	15.65	21.65	20.74	4.13	12.84	62.46	21.17	18.41
***Benzene***	0.537	1.074	0.391	0.178	0.706	0.729	0.866	1.356	0.822
***Toluene***	1.943	2.726	1.192	0.647	1.038	0.663	2.436	3.244	3.622
***CO***	0.204	0.114	0.080	0.108	0.135	0.123	0.339	0.326	0.159
***PM10***	1.38	23.93	1.03	1.83	19.68	4.64	2.76	29.67	2.62
	**Altamura, prov. of Bari (urban site)**			**Andria, prov. of Bari (urban site)**			**Monopoli, prov. of Bari (urban site)**		
	**I Source**	**II Source**	**III Source**	**I Source**	**II Source**	**III Source**	**I Source**	**II Source**	**III Source**
***NOx***	46.16	22.56	10.10	50.30	9.36	10.65	34.09	3.55	15.75
***Benzene***	0.827	0.852	0.948	0.496	0.514	0.368	0.689	0.637	0.427
***Toluene***	6.041	8.426	3.781	1.616	1.730	1.573	1.752	1.471	3.063
***CO***	0.347	0.108	0.077	0.538	0.106	0.114	0.270	0.080	0.116
***PM10***	1.47	21.93	1.34	1.70	23.54	2.38	2.20	23.89	2.50

Parameter**’**s unit is ug**/**m^3^.

Observing Table 3 and 4 that show the parameters distribution in the three pollution sources, averaged on the whole monitoring period, one can see that the profile of the second source is mostly characterized by PM_10_. The other two sources are differently characterized by NO_x_, Benzene, Toluene, CO and for a little contribution by PM_10_.

Moreover comparing the source’s profile concentrations between Summer and Winter seasons it’s possible to note a constant increasing of NO_x_ concentration from Summer to Winter for all sites and sources. In particular the first source shows for all sites bigger NO_x_ concentrations in the Winter than Summer ones. The first source can be considered a mixed source between vehicular traffic and domestic heating.

**Figure 6:**
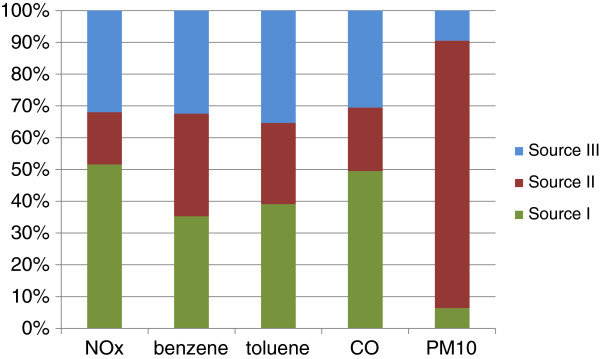
Average percentage of the parameters distribution in the three sources.

In Figure 6 the percentage distribution of the parameters in the three sources is represented. The plot is obtained from monthly sources profile averaged for all sampling period of time and among all monitoring sites.

Over 85% percent of the mass of PM_10_ is attributed to the second source. The first and third sources, composed by NO_x_, CO and aromatic compounds, and low level of PM_10_, are characterized by similar level of benzene and toluene. In particular the Toluene and Benzene concentrations ratio in the first and third sources profiles are bigger than 2 (except for San Nicola sport stadium monitoring site): in literature this value is associated to vehicular traffic emissions. Moreover NO_x_ and CO are predominant in the first source. The amount of PM_10_ in the third source, even if low, is 50% higher than first source.

These observations suggest that the second source could be identified as “Particulate source”, while the first and third sources can be considered different components of vehicular traffic emissions. In fact, no industrial plants or similar are located close the sampling sites, and the traffic is the most important source of pollution of anthropic nature. The two traffic sources might be originated by different kinds of vehicles or engines, for example gas and diesel. These different fuels are known to be responsible of different emission of pollutants. In particular diesel, before the introduction of filters, was the major source of particulate matter among the several fuels used for road transport, with lower emissions of NO_x_ and CO. Considering also the constant increasing of NO_x_ concentration from Summer to Winter for all sites and sources (Tables 3 and 4) the first source could be identified with a mixed source between vehicular traffic and domestic heating, while the third source with vehicular traffic.

**Figure 7:**
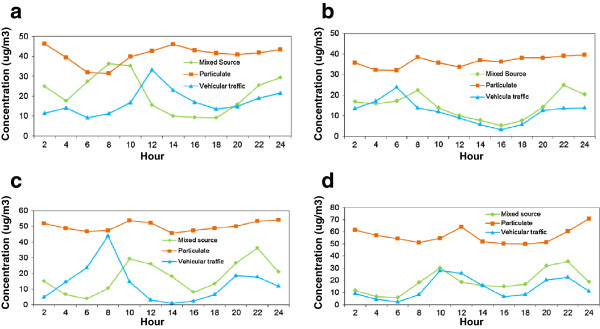
**Daily patterns for seasonal bihourly mean concentration contributions of the three sources (x axes is referred to local time: winter local time: GMT + 1 h; summer local time: GMT + 2 h) for the data collected in viale Archimede during spring (a), summer (b), autumn (c) and winter (d) 2005.**

Another proof linking the first and third sources to vehicular emissions is the daily profile of bihourly mean concentrations contributions of the three sources (Figure 7). In Figure 7 it’s clearly showed that the particulate source shows a rather constant trend during the day and it is uncorrelated with the traffic sources. The other two sources show, instead, a typical traffic profile, with peaks of emission at 8 in the morning and 20 in the evening, in correspondence of rush hours of people going back and forth from workplace. In Figure 7 the 2005 seasonal trend for viale Archimede monitoring site is shown as example: all sites have shown similar trend.

Table 5 shows the coefficients of correlation among the six sites of the three sources in the APCS profiles matrix. According to this data, we can observe that the source Particulate shows high correlation among four sites of different zones (Bari and Province). This makes our hypothesis of a regional character for PM_10_ concentrations [37]; Monopoli and San Nicola sites don’t show correlation and this can be explained considering the different nature of these sites: Monopoli is a urban sites while San Nicola is a suburban site skirting by high vehicular traffic street and whit high vehicular traffic spot during sport events (generally in the week end).

**Table 5:** Matrix of correlation coefficients of the three sources obtained by APCS sources profiles

	**Mixed source**					
	**Altamura**	**Andria**	**Monopoli**	**Archimede**	**King**	**San Nicola**
Altamura	1.00	0.07	-0.04	0.25	-0.44	0.35
Andria		1.00	-0.32	-0.02	0.44	-0.23
Monopoli			1.00	-0.06	0.05	0.32
Archimede				1.00	-0.19	0.25
King					1.00	-0.26
San Nicola						1.00
	**Particulate**					
	**Altamura**	**Andria**	**Monopoli**	**Archimede**	**King**	**San Nicola**
Altamura	1.00	***0.65***	-0.12	0.39	***0.59***	-0.12
Andria		1.00	0.10	***0.56***	***0.71***	0.13
Monopoli			1.00	***0.54***	0.05	0.24
Archimede				1.00	***0.51***	0.09
King					1.00	-0.01
San Nicola						1.00
	**Vehicular traffic**					
	**Altamura**	**Andria**	**Monopoli**	**Archimede**	**King**	**San Nicola**
Altamura	1.00	0.03	0.16	0.06	-0.07	-0.17
Andria		1.00	-0.29	0.36	0.33	0.19
Monopoli			1.00	-0.12	***0.60***	0.20
Archimede				1.00	***0.51***	0.46
King					1.00	***0.72***
San Nicola						1.00

Values higher than 0.5 are highlighted in bold text.

On the contrary, considering the vehicular traffic sources it’s possible to observe low correlation among the sites due to different location of the sampling sites.

Table 6 shows the reconstruction percentage error of the APCS model for each parameter. The error shows high variability over the range of the period. PM_10_ concentrations have shown the lowest error of reconstruction, while the CO concentrations the biggest ones. The model, in fact, suffers of low robustness when values are low (this is the case of carbon monoxide).

**Table 6:** Percentage error of reconstruction for each parameter for each site

	**Altamura**	**Andria**	**Monopoli**	**Japigia**	**San Nicola**	**King**	**Range**
***NOx***	11.13	6.99	11.22	18.16	12.14	8.81	6.99–18.16
***Benzene***	30.55	27.27	28.06	29.16	36.35	37.26	27.27–37.26
***Toluene***	9.94	11.44	8.66	21.50	15.33	11.12	8.66–21.50
***CO***	39.05	28.17	39.51	42.24	55.18	41.77	28.17–55.18
***PM10***	2.74	2.62	1.60	3.67	5.78	3.35	1.60-5.78

Anyway, in most of the cases the error was acceptable, allowing a fairly good reconstruction of the concentration trend.

### Experimental

#### ***The air quality monitoring network***

Bari is a town of about 350000 inhabitants located in South-East of Italy (latitude 41°08’, longitude 16°45’). Its greater industrial activities are in mechanical (carpentry and industrial vehicles), food and clothing sectors; its industrial area, whit a thermo electrical power station, is placed in the neighbouring towns.

Prevailing winds are from NNW and WNW in December, January and February, from East in March and September and from NNE and South in October and November. Raining days are 80 – 90 for year with maxima 40 – 50 mm. The region is characterized by an active photochemistry mostly in the summer season.

Like many other Italian cities, its urban area is characterized by high motor-vehicle traffic density, mostly in the centre of the city.

The air quality monitoring network of the Bari Municipality is composed by six fixed monitoring stations, by a mobile laboratory and a data elaboration centre. In province of Bari, that extends for 3.825 km^2^ and includes 41 towns, there are four fixed monitoring stations located in the towns of Casamassima, Altamura, Andria and Monopoli.

In this paper some stations of Bari and its province monitoring networks have been selected as representative sites of the investigated area. In Bari, the selected monitoring stations are located in residential area (viale King), in urban area (viale Archimede) and in a suburban area (S. Nicola sport stadium).

In province of Bari, the three selected stations are located in the urban and residential areas of the following towns: Altamura (67000 inhabitants) located at 47 Km south-westwards from Bari, Andria (98000 in.) at 55 Km northwards from Bari and Monopoli (50000 in.) a coastal town at 40 Km southwards from Bari.

All considered sites can be classified as urban background sites, except for Monopoli that is a urban site and San Nicola that is a suburban site skirting by high vehicular traffic street and whit high vehicular traffic spots during sport events.

#### ***The instrumentation***

Each station is provided with automatic analysers of CO (Advanced pollution Instrumentation model 300E, San Diego CA USA), BTX (model Syntech Spectras GC 855, Groningen, Netherlands), O_3_ (Advanced pollution Instrumentation model 400E, San Diego CA USA), NO_x_ (Advanced pollution Instrumentation model 200A, San Diego CA USA), PM_10_ (Opsis model SM 200, Furulund, Sweden and MP100, Environnement, France) and with meteorological sensors such as temperature, barometric pressure, relative humidity, solar radiation, speed and direction of wind and rain.

Nitrogen oxides, NO and NO_2_, were analysed using the chemiluminescence method. Measurement of ozone is based upon the capacity of such gas to absorb ultraviolet rays with opportune wavelengths, generated by built-in lamp. Carbon monoxide is analysed through the absorption of infrared rays (IR).

The measuring of PM_10_ is based upon the beta ray attenuation method on standard 47 mm membrane filters; the data are bihourly collected.

Benzene/Toluene/Xylene are measured using the capillary gas chromatographic technique in the gaseous phase, which enables the rapid separation and identification (15 minutes) of the components of the gas sample.

#### ***The data***

The data are collected by the system every hour for all parameters, except for PM_10_ that are collected every two hours. Therefore, all data are considered with means every two hours (even hours).

In order to simplify the further statistical elaborations, only days with complete data, that is days with all 12 bi-hourly means were considered for data set.

The data collected by the monitoring network was validated according to this protocol: a preliminary validation was carried out by the software, which has invalidated all data occurred in calibration hours, and data identified as artifacts; then, a manual calibration was carried out by operators, considering the relations existing among the several parameters: for example, the validation of parameters monitored by the same instrument (i.e. benzene and toluene, or the nitrogen oxides), was carried out simultaneously, like so for parameters linked by the same hypothetical source (i.e. carbon oxide and aromatic compounds, typical traffic pollutants). In this way it is possible to verify that eventual critical data are related to real pollution situations, and they are not artifacts due to instrument malfunction. Moreover, meteorological data (rain, speed and direction wind) were used to investigate about the influence of natural events on high or low concentration situation.

The data have been collected during the period of time from January 2005 to May 2007 in the investigated sites.

In the Table 1 the basic statistics for each site have been summarized.

## Conclusions

Multivariate statistical techniques such as receptor models offer a valid tool to handle complex data sets and allow to extract information not directly inferable from original data matrix by traditional approach.

In our case the model suggests that the major amount of PM_10_ isn’t linked directly to the vehicular traffic. It’s probably due to PM_10_ long and medium range transport and due to formation of secondary particulate. The model confirms a common regional contribution to PM_10_ among sites and the absence of PM_10_ seasonal trend observed.

Even if the model is applied to few parameters, it is able to suggest information about the nature of the pollution’s sources. However for the determination of the other important pollution sources, such as domestic heating, it’s needed to obtain parameters that allow to identify this source.

The results obtained by the models moreover confirm that PM_10_ concentration cannot be considered a good air quality indicator because it don’t reflect the real pollution’s sources.

## Methods

### The model description

The aim of the application of the receptor models is the apportionment of the pollutant’s sources. The two main approaches of receptor models are Chemical Mass Balance (CMB) and multivariate factor analysis (FA). CMB gives the most objective source apportionment and it needs only one sample; however, it assumes knowledge of the number of sources and their emission pattern. On the other hand, FA attempts to apportion the sources and to determine their composition on the basis of a series of observations at the receptor site only [40]. Among multivariate techniques, Principal Component Analysis (PCA) is often used as an exploratory tool to identify the major sources of air pollutant emissions [38,41-43]. The great advantage of using PCA as a receptor model is that there is no need for a priori knowledge of emission inventories [44].

PCA is a statistical method that identifies patterns in data, revealing their similarities and differences [45]. PCA creates new variables, the principal components scores (PCS), that are orthogonal and uncorrelated to each other, being linear combinations of the original variables. They are obtained in such a way that the first PC explains the largest fraction of the original data variability, the second PC explains a smaller fraction of the data variance than the first one and so forth [46-48]. Varimax rotation is the most widely employed orthogonal rotation in PCA, because it tends to produce simplification of the unrotated loadings to easier interpretation of the results. It simplifies the loadings by rigidly rotating the PC axes such that the variable projections (loadings) on each PC tend to be high or low.

Moreover the reconstruction of the source profile and contribution matrices can be successfully obtained by APCS (Absolute Principal Component Scores) method [49].

The observed pollutant concentration in the atmosphere at a certain time *C*_*i*_ can be considered as a linear combination of contributions from p sources:

(1) $$\begin{array}{l} C_{i}=\overset{P}{\underset{K=1}{\sum }}a_{\mathrm{ik}}S_{k} \end{array}$$

where *S*_*k*_ is the contribution from each source and *a*_*ik*_ is the fraction of source k contribution possessing property i at the receptor.

One of the most used methods to decompose the concentration matrix in the product of the source pattern and contribution matrices is the APCS. The starting point is the matrix X (samples × parameters). In the APCS method the first step is the search of the Eigenvalues and Eigenvectors of the data correlation matrix G. Only the most significant p Eigenvectors (or factors) are taken into account. Generally two methods are used in order to choose p Eigenvectors: Kaiser method.

Eigenvectors: Kaiser method (PCs with eigenvalues greater than 1) and ODV80 ones (PCs representing at least 80% of the original data variance).

The p Eigenvectors are then rotated by an orthogonal or oblique rotation. The most used rotation algorithm is Varimax, which performs orthogonal rotation of the loadings. After the rotation all the components should assume positive values; small negative values are set zero. An abstract image of the source contributions to the samples can be obtained by multivariate linear regression:

(2) $$\begin{array}{l} Z=\mathrm{PC}S^{*}V^{T} \end{array}$$

where *Z* is the scaled data matrix, *PCS* is the principal component scores matrix, and *V*^*T*^ is the transposed rotated loading (Eigenvectors) matrix.

In order to pass from the abstract contributions to real ones, a fictitious sample Z_0_, where all concentrations are zero, is built [43,50]. Details about the method can be found in the reference 49: the APCS matrix can be identified with the estimated contributions matrix *F*_*r*_. A regression on the data matrix *X* allows to obtain the estimated source profiles matrix *A*_*r*_. At last the product of the matrices *F*_*r*_ and *A*_*r*_ allows to recalculate the data matrix *X*_*r*_ (reconstructed data matrix). The reconstruction percentage error of the model has been calculated as percent relative root mean square errors (RRMSE) as shown in reference [49].

The authors declare no experimental research has been performed on animals or humans in the frame of the research activities related to this paper. No ethics committee exists for this kind of research.

## Competing interests

The authors declare that they have no competing interests.

## Authors’ contributions

PI carried out statistical elaborations, results interpretation and coordination the drafting manuscript. VP carried out data analysis, statistical elaborations and results interpretation. He gave contribution to drafting manuscript. GdG carried out the coordination of the research, participation in its design and results interpretation. PRD performed data analysis and coordination of the study activities. All authors read and approved the final manuscript.

## Authors’ information

1Researcher at Water Research Institute - National Research Council, Bari, Italy 2Researcher at Chemistry department, Bari University, Bari, Italy.
